# Evolving Conceptions of Work-Family Boundaries: In Defense of The Family as Stakeholder

**DOI:** 10.1007/s41463-022-00124-6

**Published:** 2022-03-22

**Authors:** Miguel Pina E. Cunha, Remedios Hernández-Linares, Milton De Sousa, Stewart Clegg, Arménio Rego

**Affiliations:** 1 Universidade Nova de Lisboa, Lisbon, Portugal; 2 Universidad de Extremadura, Mérida, Spain; 3 University of Sydney, Sydney, Australia; 4 University of Stavanger, Stavanger, Norway; 5 Católica Porto Business School, Porto, Portugal; 6 Instituto Universitário de Lisboa (ISCTE-IUL), Business Research Unit (BRU-IUL), Lisboa, Portugal

**Keywords:** work-family, stakeholder theory, family as stakeholder, COVID-19

## Abstract

In the management and organization studies literature, a key question to explore and explain is that of the family as an organizational stakeholder, particularly when working-from-home became the “new normal”. Departing from meta-analytic studies on the work-family relation and connecting with scholarly conversation on work-family boundary dynamics, we identify three main narratives. In the *separation narrative,* work and family belong to different realms, and including the family in the domain of organizational responsibility is seen as pointless. The *interdependence narrative* stresses that organizations and families are overlapping domains in which it is important to acknowledge that the policies and practices of the former might have an impact on family life, and vice-versa. The *embeddedness narrative*, brought to the fore by the COVID-19 pandemic, sees employment and family as progressively convergent and hybrid work domains. The evolution of employment relations towards increased hybridity of the work situation being embedded in the familial/household context increasingly calls for consideration of the family/household as an integral rather than a peripheral stakeholder.

## Introduction

“While families have a large and undeniable impact on human behavior, management research is yet to fully embrace how aspects of families (e.g., family-member relationships, family structures, and family events) influence entrepreneurs, employees, managers, and their organizations.”

Jaskiewicz et al. (2017, p. 309).

Organizations enact and participate in a great many networks of stakeholder relations (Freeman 2010). From a stakeholder perspective, “business is seen as a set of relationships among groups that have a stake in the activities that make up the business” (Parmar et al. 2010, p. 405). In a typical formulation, stakeholders include “employees, suppliers, customers, communities, civil society, governments and others” (Böhm and Pascucci 2020, p. 548) that interact to jointly create and trade value. According to this perspective, “It is the executive’s job to manage and shape these relationships to create as much value as possible for stakeholders and to manage the distribution of that value” (Parmar et al. 2010, p. 406). Managing and shaping relationships with stakeholders is both an economic and moral endeavour. However, with some notable exceptions (Mitchell et al. 2011; Venter et al. 2012), the family is not included in lists of stakeholders (Friedman and Miles 2002). In management and organization studies (MOS), the family is often seen as part of a “non-work” arena, a “haven in a heartless world” (Lasch 1995). As an extra-organizational source of identity, family is often seen as an attribute akin to religion, gender, or nationality (Ramarajan and Reid 2013).

At the same time, the family has been touted as a core institution in human life (Laslett 1973), and it has been claimed in prior research that the family should be considered as “the missing variable in organizational research” (Dyer 2003, p. 401). Recent research, building on Morgan (1975, 1996, 2014), stresses the family less as an institution (Parsons, 1951) and more as a site of social practices and lived family experiences. As such family practices are a form of social organization with a multiplicity of expressions, institutionally regulated by law, norms, religion, etc. (Rogers 2017). Seen thus, as a form of organization, then the family can be seen as existing in a tense relationship with other organizations that make significant claims of one’s time, such as employing organizations (Kopelman et al. 1983; Michel et al. 2009), namely because time spent at work in an organization cannot be time spent with the family as a social organization. The mundane idea of “going to work” suggests this separation succinctly; work is *another* place.

Historically, the workplace was part of the public sphere and coded as masculine, while the organization of the family was a private space, coded as feminine; these distinctions were embedded in classic statements such as the separation of the public and private spheres in Weber (1978). Organizations and their practices were clearly a public space whose resources and affordances should be sequestered from the private sphere. Nonetheless, in the twenty-first century, as Casper et al. (2018, p. 182) put it, “Everyone seems to want work–life balance”, which entails a blending of the masculine and feminine realms of work and family as normatively conceived. As they go on to say “Anne-Marie Slaughter ignited controversy when she argued that work–life balance is not possible for women, who cannot ‘have it all’ (Slaughter 2012)”. Given the importance of this interface as well as its’ highly visible consequences, how can the absence of the family as an organizational stakeholder be interpreted?

The exception of the case of the family not being considered in the management and organizations literature is the family-owned business (Uhlaner et al. 2004; Zellweger and Nason, 2008). Elsewhere, the absence of discussion of the family as a social organization with a stake in the sphere of formal organization is in part historical. A dualistic interpretation of family and work has lineages embedded in nineteenth century bourgeois assumptions that coded the workplace as a masculine controlled public sphere while domestic space was private and under command and control by the lady of the house, a distinction inscribed in law (Baron, 1981). Given this legacy, family and work existed in separate spheres, as Gerson (2004, p. 163) notes:

“A half century ago, when most women withdrew from paid work to rear children and men’s breadwinner status went largely unquestioned, work and family life were generally conceived as ‘separate spheres’ (Acock & Demo, 1994). In the closing decades of the twentieth century, as women joined the labour force in ever mounting numbers and gender boundaries began to blur, ‘work and family’ emerged as a distinct field of study and the image of work–family conflict gradually but inexorably replaced long-held assumptions about ‘separate spheres’ for women and men (Barnett & Rivers, 1996)”.

With these major social transformations, opposing work and family as different realms, with the family realm lying outside the scope of MOS, is a convention difficult to sustain and one whose applicability has been greatly changed by the work from home strategies adopted during the COVID-19 pandemic. Indeed, the COVID-19 pandemic confounded oppositions between work and home as separate places in which family-work conflicts centred on time spent on different pursuits in different places (Greenhaus and Powell 2006) became instead a feature of a coterminous space. With responses to the pandemic, micro and liminal “recurring transitions that occur on a frequent basis” (Allen et al. 2014, p. 101), such as daily commutes, disappeared and challenged notions of work-family balance (Pradies et al. 2021). The pandemic interrupted this form of daily “border crossing”, as it collapsed physical, temporal, and psychological domains into one. Based on these opening remarks, we investigate the work-family boundary literature to discuss two related research questions: (1) *how has the family as an organization been considered (or not) as a stakeholder in formal organization?* (2) *what does COVID-19 reveal about family-work tensions?*

To do so, we advance a comprehending (Sandberg and Alvesson 2021), paradoxical (Smith and Lewis 2011), and non-dualistic (Tsoukas 2019) view of family organization as a formal organizational stakeholder. Comprehending theories are meaning-making conceptual systems that contribute to ongoing theoretical conversation by redirecting attention to some specific topic. In our case, accepting that work and family exist in a state of tension, with paradoxical characteristics of opposition and persistence (Smith and Lewis 2011), we suggest that the COVID-19 pandemic “visibilized” (Tuckermann 2019) work-family embeddedness, and we explore the implications of this change for the consideration of the family as an organizational stakeholder. We suggest that developments in technology, work and organization, such as the diffusion of telework and the home office, working from anywhere (Choudhury et al. 2021; Kniffin et al. 2021; Kurland and Bailey 1999), flex time (Gonsalves 2020), and home-based businesses (Reuschke and Mason 2020) render this topic especially relevant.

With this article we contribute to discussion of work-family tension by suggesting that the family may constitute itself as an organizational stakeholder rather than being an extension or “the other side” of employees’ lives. The idea that the family as a form of social organization is a stakeholder in formal organization is one that stakeholder theory has not entertained; hence, our contribution addresses a significant gap in the MOS literature related to organizations and stakeholders. Our proposal is based on the progressive intertwinement of the two realms, as will be discussed next.

## Reasons for Marginalizing the Family in Stakeholder Theory

In this section we discuss how and why the family has been marginalized in stakeholder theory’s increasingly longer list of stakeholders. We consider three main reasons for this conceptual neglect: (1) narrow conceptions of the worker, (2) dualism, and (3) methodological individualism, i.e., the methodological propensity to study individuals. MOS has contributed enormously to our understanding of organizational functioning, but it has been bounded by its onto-epistemological assumptions. We suspect that the assumptions we present here are common to other theories of organization (e.g., Tsoukas 2019).

First, the worker has often been represented as a *homo economicus* (Doucouliagos 1994) rather than as a whole person that exists beyond the organization’s boundaries. Even when the worker was represented as a being social, emotional and spiritual, such representations have focused mainly on the work domain or on their effects on the work domain. Second, dualistic separation is often used in MOS, as in the work vs. non-work divide. For example, significant attention has been devoted to work identities (e.g., Ashforth and Kreiner 1999) but less attention has been paid to the overlaps and negotiations between work and non-work identities (e.g., Ramarajan and Reid 2013) and, especially, the way work and family processes and identities coexist. Third, the extensive methodological individualism of the organizational behaviour corpus (Hosking et al. 1995) biases a focus on subjective personal motivation as appropriate for studying entities such as persons, rather than focusing on processes, relationalities, and interactions (Tsoukas 2019).

### Narrow conceptions of the worker

MOS rested upon a work and non-work separation assumption. In foundational texts (Taylor 1911), people were employees being made into experts in obedience (Jacques 1995; Watkins and Dalton 2020), minimizing their reflective and agentic powers as people who strove to preserve their energies in work for multiple other life domains (Clegg et al. 2006). The construction of the individual as obedient employee is manifest in many ways. The process of the social construction of the employee, as told by Jacques (1995), depicts workers as tools, “human resources” over whom, in an extreme case, with the creation of Ford’s Sociological Department a form of moral surveillance of employee’s family lives became normalized (Clegg et al. 2006). The agency of being at work determined the appropriate being not at work, at home and in other private spaces.

Such historical legacy, combined with the cognitive view of organizations (Brief and Downey 1983), fundamentally conceptualized individuals as independent, rational beings who could be trained and drilled in work to manage other spaces with a rationality that their employers could reward. Stakeholder theory softened some of the hard edges of these earlier conceptions by suggesting that employees were “stakeholders” (Friedman and Miles 2002), independent contributors bringing meaning from their life worlds to work. The emotional turn of the 1980s (Fineman 2000) and a growing awareness of the role of relationality in organizations (Cooper 2005; Uhl-Bien 2011) meant more attention was paid to affect and emotion in the workplace yet, always within boundaries defined by the organization.

### Dualism

Dualism refers to doubleness, defined as the existence of a relationship of opposition and conflict between two elements (Farjoun 2010) such as work and family. It is well established, for example, that organizational policies and practices often constrain individuals in their family roles (e.g., Kopelman et al. 1983; Zhao and Mattila 2013). As theorized by dualism, the two are in tension but their relationship is fundamentally dilemmatic: gains in one domain represent losses in the other and while managers have responsibilities in one domain they do not in the other. In MOS, the relationship between work and family has largely dealt with this dualism, as in the influential work of Edwards and Rothbard (2000, p. 180) by theorizing “linking mechanisms” between work and family constructs. The two domains have been conceptualized and investigated as distinct (Kabanoff 1980).

### Methodological individualism

Methodological individualism refers to understanding organizational behaviour in terms of individual acts (Tsoukas 2019) rather than the relationships that support human flourishing (Dierksmeier 2016). As noted by Pirson et al. (2021), MOS has been pervaded by an individualistic approach, sometimes taken to the extreme. Individual acts are subsequently aggregated at other levels of analysis (e.g., teams, organizations, networks, and even societies). Those higher-level populations are interpreted as aggregates rather than relations and interactional units. The family and its collective dynamics in the context of organizations are rarely a habitual unit, except in the unique case of family business (Calabrò et al. 2018).

## Marginalization is Unsustainable from a stakeholder perspective

The COVID-19 pandemic and the lockdowns that forced millions to work from home (Hemmings 2020; Kniffin et al. 2021) suggest that it is timely to reconsider the family as a legitimate and important organizational stakeholder. The pandemic accelerated trends already underway: some offices were becoming more like home through practices such as the adoption of pet policies (Cunha et al. 2019) and by designing living spaces in the workplace (e.g., gaming and relaxing areas, gardens, work environments designed similarly to living room, etc.). Meanwhile, in a sudden shift, homes became more like offices as the pandemic struck and people worked from home. Children and pets (Kelemen et al. 2020) were no longer hidden in a private space but visible to peers and managers. Organizations, recognizing how family life and work were becoming increasingly entangled in a difficult and nervy balancing act, offered support in the form of mindfulness and wellbeing sessions. Therefore, the pandemic made apparent that the family was not a marginal factor, adjacent to yet separate from work but a form of organization made integral to formal work and organization. It is thus important to explore how MOS literature has, or has not, recognized family as an important stakeholder and how the pandemic accelerated such a trend.

## Method

We followed an inductive and interpretive approach to theorizing, our main objective being understanding of why family organization has not featured as a stakeholder for formal organization in MOS. Conceptually, we explore some of the possible implications of theorizing family organization as a stakeholder. We followed the methodological recommendations of inductive research (Böhm and Pascucci 2020; Gioia et al. 2013) and used the existing literature to compose conceptual propositions, forming a three-scenario theoretical framework on the family as a stakeholder.

In line with Böhm and Pascucci (2020), instead of conducting a traditional literature review, we established a coherent narrative related to the evolution of the work-family relationship by purposefully sampling published meta-analytical works on the role of the family in MOS. We proceeded in two steps. First, to develop a broad understanding of the literature, we identified meta-analytical works by performing a systematic search in *Web of Science (WoS)*, a comprehensive citation database characterized by its objective scholarly journal selection standard, as well as by its widespread diffusion within the scientific community (Hernández-Linares et al. 2018; Perri and Peruffo 2016). Specifically, on October 20, 2021, we searched all “articles” and “reviews” (published in English) in which *family*, *work,* and *meta-analysis* or *metanalysis* appeared as topics. These keywords were accompanied by special characters to capture any orthographic and/or linguistic variation (a common practice in the field: see, for example, Weale et al. 2020).

We used the entire *WoS* database to avoid any omission or potential bias derived from considering only a set of relevant journals (López-Fernández et al. 2016). However, we limited our search to published (or forthcoming) articles and reviews published (or in press) in academic journals. It is common to do so in literature reviews (e.g., Byron 2005; Zhao et al. 2020a) because publications in peer-reviewed journals are considered validated knowledge which has the largest impact on scholarly discourse (Kossek and Ozeki 1998; Podsakoff et al. 2005). As result of our search process, once duplications were eliminated, we identified 853 documents, which had been published in 123 different *Web of Science* categories, with “Medicine General Internal” (103 documents), “Public Environmental Occupational Health” (91), “Psychiatry” (89), “Psychology Applied” (72) and “Management” (70) being the categories with more studies published, the remaining below 45 studies each. The search revealed a growing scholarly interest in the topic: until 2008 the number of works published per year remained below 20, in 2015 they reached 54 and this number was doubled in only 5 years (108 publications in 2020).

We then conducted an inductive, qualitative analysis of the titles and abstracts of these 853 documents, to eliminate both misclassifications and papers not focused on the study of the family-work relationship. Although the selected time limit was the maximum allowed to prevent distortion of the results (Hernández-Linares and López-Fernández 2018), the first meta-analysis found was published in 1998 by Kossek and Ozeki. Then, we complemented this search with a review of key exemplars that helped us make sense of the impact of the COVID-19 pandemic over organizations and organizational behaviour (Kniffin et al. 2021). The meta-analytic sources included 34 studies (see Table 2 in appendix 1) focused on the dynamic relations between work and family dimensions, which were published in 20 journals (see Table 3 in appendix 2).

Analytically we ordered the literature by selecting representative narrative templates and then abstracting these first-order templates to gain theoretical distance. These works revealed that the extant literature paid special attention to work-to-family and family-to-work conflict, explaining the reasons why these domains have been mainly seen, in recent decades, as a field of tension, after an initial period of separation. From these sources, which we complemented with others that were theoretically sampled and mainly COVID-19 related, we derived a model with ordering intentions (Sandberg and Alvesson 2021), i.e., developed to assist scholars making sense of the evolution of the field. Doing this allows us to consider past and present trends, namely how the COVID-19 pandemic is redefining the boundaries of work and family.

**Figure 1:**
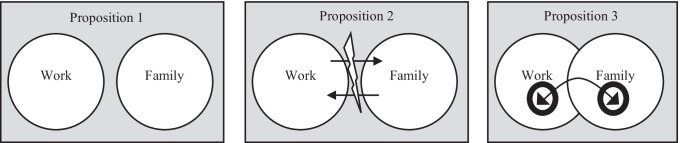
Three propositions on the work-family relationship.

By interpreting these works inductively, we composed three coherent scenarios or conceptual propositions on the work-family narrative: separation, interdependence, and embeddedness (Table 1). Our three narratives incorporate prevailing analyses and theorizations but also include recent developments triggered by the pandemic. It is known that pandemics potentially have long-lasting changes in terms of the representation of the workplace (e.g., Fayard et al. 2021) and there is no reason why the present case should differ. In this sense, our work combines an effort to zoom in and out (Nicolini 2009): we zoom in on existing research and zoom out from it to see how the pandemic raised new questions at the boundary between work and family. We develop three propositions (depicted in Fig. 1): (1) *work and family constitute separate domains (or the separation narrative),* (2) *work and family are overlapping domains (interdependence narrative), and* (3) *work and family are embedded (embeddedness narrative).* We do not consider that our scenarios exhaust interpretive possibilities. Other approaches such as differences between family and non-family businesses, dual and non-dual work couples, as well as differences between the knowledge work elite and the “cybertariat” (Burrell and Fourcade 2021) might have been chosen as a basis for analysis. We have chosen to consider the tensions between work and family domains of organization as our conceptual focus. Different conceptual angles may certainly produce different scenarios, a possibility that might be explored in future research.

**Table 1:** Work and family: logics, tensions, implications.

Exemplary narratives from the literature	Key tensions (organizational view)	Dominant logic	Implications for management and for the stakeholder view
“In everyday life, psychological detachment from work is experienced as “switching off’ and means leaving the workplace temporarily behind oneself in physical and in mental terms.” (Sonnentag et al. 2010, p. 965) “segmentation of work and family life is a phenomenon that emerges in part as a function of industrialization and economic growth. This likely coincides with the separation of employees from their elders, who have greater means to retire independently. Thus, it may be that the expectation for segmented work and family roles is less pronounced in economies with low-level economic development (Xu et al. 2018, p. 262) “Rooted in role theory, and derived from a scarcity hypothesis (fixed amount of resources, such as time and energy), conflict theory posits that the work and family domains can be incompatible resulting from different norms and requirements (Burke, 1986; Evans and Bartolome, 1984; Zedeck and Mosier, 1990); thus, increased role performance in one domain (such as work) results in decreased role performance in the other domain (such as family):” (Michel et al. 2009, p. 200) “Lower levels of individualism and economic development may be associated with a more integrative view of the work–family interface, with a greater priority placed on the resources work provides for one’s family and the extent to which work helps fulfill obligations to family members” (Xu et al. 2018, p. 262) “One regional partner and his wife reported that they could not understand why members of the firm sought to segregate their professional from their personal lives. For this couple, the professional life was the personal life and, for them, this melded existence was “fun.” Spouses were expected not only to represent the firm at such events as client functions, but also with the firm member to whom they were married. The regional managing partner who described the use of flip charts at monthly meetings proudly stated that he sent the entrepreneurial reports home to the partners’ spouses “to add a little more pressure” for achieving the individual’s, office’s, and region’s objectives. Thus did norms and normalization extend from the professional to the personal life as inspections became more meticulous, even fussy.” (Covaleski et al. 1998, p. 312)	(1) Work and family are different domains. Interference should be minimized... …but (2) The organization may interfere instrumentally when appropriate.	Logic of separation: Work and family are distinct and independent domains Ontological stance: Dualism (work and family as separate domains)	Family as non-stakeholder: Family is outside organizational reach. It belongs to the private domain. Outside organizational jurisdiction.
“home life can become seriously depleted when both men and women work long hours. As households and families are starved of time, they become progressively less appealing, and both men and women begin to avoid going home. (...) for many professionals, “home” and “work” have reversed roles. Home is the source of stress and guilt, while work has become the “haven in a heartless world” – the place where successful professionals get strokes, admiration, and respect.” (Hewlett and Luce, 2006, p. 55) “the influence of the work and the family domain is reciprocal and should be seen as a spiral rather than a unidirectional process (Demerouti et al., 2004).” (Amstad et al. 2011, p. 162) “positive reciprocal influences of work and family (work–family enrichment or facilitation) have been demonstrated in a number of studies (see Greenhaus and Powell, 2006).” (Amstad et al. 2011, p. 163) “The interface between work and family has received broad attention during the past 20 years. Research interest in this topic is associated with changes in societal structure, especially the rising number of dual-earner couples with children. This interest in the challenge of combining work and family is not likely to fade in light of foreseeable changes in the family as well as the work environment for a number of reasons. Regarding families, the number of dual-earner couples with children is not likely to decline. Therefore, more and more individuals have to combine work and family responsibilities. Second, child care is no longer exclusively a women’s topic because fathers’ involvement with children is growing (Halpern, 2005). Third, the number of single parents is rather high, which might have an impact on combining work and family duties (Duxbury et al., 1994). Fourth, external child care will likely become more common and even perhaps be taken for granted, which allows parents more control over their family duties and possibly facilitates combining work and family responsibilities (Voydanoff, 2005c).” (Amstad et al. 2011, pp. 163–164) “the present meta-analysis demonstrates that work–family conflict affects well-being and behavior not only in general, but also with respect to family and working life. However, it is important to stress that combining these two life domains can have a positive effect as well. It has repeatedly been demonstrated that living multiple life domains has a positive effect on individuals’ wellbeing and health (Barnett and Hyde, 2001; Kotler and Wingard, 1989; Ross and Mirowsky, 1995). Furthermore, positive reciprocal influences of work and family (work–family enrichment or facilitation) have been demonstrated in a number of studies (see Greenhaus and Powell, 2006).” (Amstad et al. 2011, p. 164) “if women themselves prefer to be with their families, as the work–family narrative has it, leaders cannot be accountable for the glaring gender inequality in their senior ranks. Nor do they need to confront the disturbing possibility that they themselves might be biased or might have discriminated against women. Nor need women, for their part, confront the possibility that they might have been in any way ill-treated or victims of discrimination. To the contrary, in the course of detailing the work–family account, many participants of all ranks and both sexes went to great lengths to assure interviewers that women’s lack of advancement could not be the result of discrimination, suggesting that this unpleasant possibility existed, at some level, in their consciousness. The defense system, however, ensured that it was never seriously broached.” (Padavic et al. 2020, p. 98) “Work–family conflict and couple relationship quality appear to be more closely linked for single-earner couples than for dual-earner couples. Perhaps this is because dual-earner spouses can relate to their partners’ struggle to balance work and family demands and therefore are more sympathetic when work interferes with family life. It could also be a reflection of the fact that dual-earner couples are both more work-centric, so concerns about work are less likely to interfere with their relationship. This finding supports the work of Yogev and Brett (1985), who suggested that symmetry in level of role involvement yields positive outcomes for couples.” (Fellows et al. 2016, p. 514)	(1) Find work-family balance… … but (2) Unbalance the balance when instrumentally advantageous.	Logic of integration: Work and family coexist, overlap, and frequently collide Ontological stance: Duality (work and family as mutually constituted domains) Factors: dual career couples, gender equality, change towards knowledge work and telework	Family as peripheral stakeholder: Family and work interpenetrate
“About half (55%) of US companies allow employees to work at home occasionally and one-third allow employees to work at home or off-site on a regular basis (Galinsky and Bond, 1998). About one-fifth of all employees report working some of their regularly scheduled work hours from home (Bond et al., 1998) and approximately 24 of the 65 million employed adults who use a computer to perform their job, do some of their work from home (US Department of Commerce, 2002). There are between 13 and 19 million workers in the United States who work at least one day a week from home during regular business hours (Kossek, 2001).” (Hill et al. 2003, p. 221) “Although availability of flextime policies are likely to provide the employee with a sense of control (Kossek et al., 2006; Thomas and Ganster, 1995), actual use of policies may increase control or, in the case of involuntary use (e.g., being assigned to telecommute), decrease control. The assumption seems to be that being in a flexible work situation is desirable. However, given a choice some employees may prefer traditional work schedules and locations.” (Allen et al. 2013, p. 362) “work and family life are both clearly more susceptible to intrusions when conducted in the same location (Ahrentzen, 1990).” (Standen et al. 1999, p. 374) “organizational support for employees’ family and life demands is more likely to play critical roles in individual family and life attitudes rather than work attitudes” (Zhao et al. 2020b, p. 3776) “Therefore, we agree with other researchers that organizational work–family interventions should also target family members of the employee (Green et al., 2011; Matthews et al., 2006). For example, organizations may offer counseling for couples who struggle with balancing work and family needs. Organizations can also increase the involvement of partners by soliciting their perceptions of organizational cultures and practices.” (Li et al. 2021, p. 97). “the results here support the general conclusion that the work-family enrichment has benefits for both work and family life. As such, organizations can attempt to improve employee well-being and performance not only through the reduction of work-family conflict, but also through the enhancement of work-family enrichment” (Zhang et al. 2018, p. 224)	(1) Regulate embeddedness… … but (2) Define the rules and keep the last word.	Logic of embeddedness: Work and family are mutually embedded Ontological stance: Dialectics (work and family as mutually transforming domains through conflict)	Family as integral stakeholder

As noted in Table 1, in the first case the family is a non-stakeholder, in the second, a peripheral stakeholder, and in the third an integral stakeholder. The Table presents evidence of each approach, drawn from the literature.

### Proposition 1: Work and Family Constitute Separate Domains (Separation Narrative)

In MOS, in the past work and non-work were devised as distinct realms. Even though they existed in tension, they were taken as separate. Ontologically, the relation was one of dualistic separation. This is captured, for instance, in the notion of psychological detachment, in which, while at home, people mentally disconnect from work (Sonnentag and Bayer 2005). The narrative is one of opposition, even if spill-over effects occur from one domain to the other.

Organization theory has traditionally treated the family as laying outside its domain of inquiry. The family could be considered relevant for disciplines such as sociology (Greenfield 1961), anthropology (Kertzer 1984) or family studies (Hamon and Smith 2014), but not in MOS. The relation of work and family is represented here as of the either-or type, a prevailing logic in MOS, in which one process and its opposite tend to be viewed as existing in a state of opposition and separation (Farjoun 2010). As an independent phenomenon, the family has not been traditionally engaged by organization theorists, even though there is necessarily a degree of permeability between the two domains. Meanwhile, outside the MOS domain, the shift from conceptualizing the family as an institution to thinking of it as an organization with a multiplicity of forms (Rogers 2017) makes the importance of rethinking these relations evident. Social and formal organizational relational domains are entangled (Barling and Macewen 1992) but mainstream MOS scholarship’s interest is largely in only one organizational side: that of the employing formal organization, not the social organization of the family. Dealing with the work-organizational domain, the private side, including the family, can be marginalized by MOS.

Scholars have been aware of work-non work inter-role conflict and work-non work boundary dynamics. While Kanter (1977) famously criticized the “myth” of separate worlds, work-family overlaps were mainly viewed from the perspective of work, as in the case of expatriate failure because of the spouse (Gupta et al. 2012) or the loss of female talent because of family life and female dropouts (Cech et al. 2011). For the family side, there was a specific, dedicated field, that of family studies. From the organizational side, family emerged as a source of competition for time (Hewlett and Luce 2006), work-life balance (Clark 2000) and more freely, coordination of dual couple agendas (Petriglieri 2019). Most of these works emphasize the trade-offs involved in the process of articulating work and family. In this narrative, as in Whyte’s famous formulation, the organization man “belonged” to the organization (Whyte 1956; see also Randall 1987), despite, as Covaleski et al. (1998) found, that the spouses of these organizational men were contingently co-opted to support their work (see representative quote in Table 1). As noted by Covaleski et al. (1998), managers in the big accounting firm they studied added to the pressure that employees felt by engaging with employee’s spouses. Important contributions were produced from this stream of work highlighting, for example, the hegemonic structures preserving prevailing work cultures (Padavic et al. 2020) and the costs associated with sacrificing personal needs to fulfil corporate requirements (Gratton and Ghoshal 2003).

The previous focus is understandable, given that organizational behaviour deals with *organizational* contexts. What lies outside the formal context of the organization as a work-place space is not within the domain of MOS. In this perspective, workers are independent individuals, which explains the reluctance to study supra-individual units, such as dyads (Tse and Ashkanasy 2015) and relationships (Cunha et al. 2015). Despite the growing recognition of organization as relational space (Dutton 2003), the focus on workers as individuals being at work is dominant. The fact that these individuals have identities forged in other enduring forms of organization, such as their family and household form, is discounted. Research in this tradition has centred on the internal side of the organization. Knowing that family boundaries are more permeable than organizational boundaries (Hall and Richter 1988), however, there is one clear tension: the organization does not interfere with the family unless it is instrumentally advantageous to do so.

### Proposition 2: Work and Family are Overlapping Domains (Interdependence Narrative)

A second stream approaches work and family as integrated domains. Instead of being represented as independent or only episodically overlapping, work and family became mutually influencing, co-existing as a dynamic balance between persistent, interdependent domains, as a paradox (Berti et al. 2021; Smith and Lewis 2011). It is because the domains of work and family can be represented as composing a paradox that the persistent tension between them can be navigated but not resolved (Cunha and Clegg 2018). Ontologically the relation is one of duality (Farjoun 2010). The decline of the era of the “organizational men” (Whyte 1956) was catalysed by the advent of dual career couples (Kanter 1977).

Dual career couples brought new motives, arrangements and challenges to the domains of work and family. Work was changing and with it the workers: they were no longer viewed as the docile employees of the past, especially in knowledge intensive firms (Starbuck 1992). They were now portrayed as more mobile and flexible (Gratton and Ghoshal 2003). The relation between work and home was characterized by opportunities that benefitted both work and family (the enrichment thesis; Greenhaus and Powell 2006) as well as by costs created by conflicts over resources, such as time and attention (Voydanoff 1988). The interdependence narrative fundamentally focuses on the role and notably, the salience, of conflict. It attempts to find a balance between the two domains.

The interdependence narrative emphasizes not the search for a solution but attempts to find some state of paradoxical dynamic equilibrium (Smith and Lewis 2011) in which workers continuously split *and* integrate work and family as opposing but mutually defining forces. Such balance can be obtained from the family side (via flexibly working from home) and from the organizational side (via family-friendly policies). The paradoxical nature of the relationship renders the endeavour difficult, as reaching and maintaining balance is challenging. Padavic et al. (2020) observed empirically that even family-oriented policies, intended to favour women, may aggravate the lack of balance. The persistent lack of balance is indicative of vicious circularity, a repetitive dynamic in which attempts to solve a problem end up aggravating it, in a self-reinforcing dynamic that is difficult to grasp and to interrupt (Tsoukas and Cunha 2017).

### Proposition 3: Work and family are embedded (embeddedness narrative)

The logic of embeddedness emerged from the departure from traditional forms of work and the workplace. In 1980 Alvin Toffler predicted that work would be relocated from the employer’s facilities to the employee’s premises. Toffler (1980) anticipated that jobs will shift from the factory or office to “where they came from originally: home” (p. 210), albeit that it has taken forty years and a global pandemic to validate the prediction although working from home was promoted in the 1980s by Californian companies such as Yahoo (Messenger and Gshwind 2016). More recently, the old representations of work and home as separate domains are giving way to new forms of work-family articulation that are in the process of coming into being. One impetus has been technological. Increasingly sophisticated digital devices created a progressive embeddedness of work and home via an “always on” type of culture (Kelly and Moen 2020, online).

The major impetus has been epidemiological as new possibilities for virtual organizing were boosted by the COVID-19 pandemic. The result has been the normalization of so-called “hybrid workers” who work partly at home, partly in the office (Fayard et al. 2021). The traditional bipolar spatial structuring of work is challenged, suggesting that in the future work would be detached from some specific space (the “workplace”) and that it could be done from anywhere (Kurland and Bailey 1999), including home (Standen et al. 1999). As an illustration, during the COVID-19 pandemic, Twitter announced that workers could work from home “for ever” (*Financial* *Times* 2020, p. 10). In another example, the insurance company Liberty decided to allow employees in Europe to work only from home, offering an additional 660 Euros annually to cover expenses (*The Irish Times* 2021). The COVID-19 pandemic constituted a massive experiment with remote work, with recent studies showing that productivity in some cases increased during the pandemic (Barrero et al. 2020; Bloom et al. 2015; Choudhury et al. 2021). At the same time, companies realised that they could save substantially on real estate costs, with many revising their understanding of the office (Fayard et al. 2021).

New technological possibilities and the changing nature of work as knowledge work (Blackler 1995) popularized new work arrangements, such as teleworking or working from the home office. That employees might have difficulties in maintaining boundaries between work and non-work (Ramarajan and Reid 2013) was accentuated by the pandemic. It is possible that habituation to the new form will create tension with the old form to create a new synthesis, as explained in dialectics (Hargrave and Van de Ven 2017), the ontology of this approach. We do not attribute positive or negative characteristics to such synthesis; while it will certainly bring positive possibilities, it will also open new possibilities in modes of monitoring and surveillance (Bhave et al. 2020; Zorina et al. 2021), which explains the need for new legal frameworks for remote work.

Emerging forms of work become “increasingly intrusive into the time and space normally reserved for personal life” (Messenger and Gshwind 2016, p. 205). At some point, these domains entangle and leach into each other, work and family organization becoming permeable, making it difficult to clearly disentangle one realm from the other. Managers are urged to learn from successful marriages (Alony 2020) and to conduct “learning by living”, including parenting, which is presented as a template for leadership development (Sturm et al. 2017, p. 364). The family-work relationship becomes increasingly dialectical, displaying characteristics of tension, reconciliation, and synthesis. It is becoming clear that, as people spend more time in the home office, family organization becomes integral to the other organizational landscape. Assuming that family organization is no longer removed from MOS’ conceptual bounds, it seems realistic to consider that the once private space is becoming part of organizational networked space, part of a distributed office, articulating multiple physical territories, including the hybrid office, the home office and the anywhere office.

Research indicates that working from home has many positive benefits, such as less partner conflict, better monitoring of children, as well as flexible schedules that may provide benefits in responding multiple domains (Hill et al. 2003). For this reason, organizations might want to keep the benefits of giving people more freedom and choices in hybrid organization of work. Instead of designing “one size fits” all solutions*,* policies that integrate distinct needs of different segments of the workforce (i.e., different jobs and tasks, different *projects and workflows, different employee preferences*) become more appealing (Gratton 2021). People in different life stages have different needs (Ahlrichs 2007), indicating diverse work-family arrangements for different occupational segments. Not only young children’s parents but also employees with aging parents have needs that such arrangements might meet. More flexibility on the part of organizations may be an expression of social responsibility as well as an increasingly salient factor for talent retention.

Well-designed but flexible and customized options may serve to empower people to make work decisions and to mitigate some negative management practices, projecting harmful consequences, namely for health (Goh et al. 2019). For these reasons, the hybrid workplace, promoting family organization as a stakeholder, may deter inadequate management practices and create mitigating conditions for some damaging effects of the modern workplace, including close monitoring (Gonsalves 2020) and presentism (Padavic et al. 2020), even when flex time and work-life balance are proclaimed values.

## Discussion

We make three major contributions to the work-family literature by stressing the role of family organization as a stakeholder rather than seeing it as in normative institutional terms. First, the inescapability of tension; second, the progressive approximation of work and family beyond family firm literature; third, the need to treat forms of family organization as legitimate stakeholders, especially in face new forms of work, widely experienced during the pandemic.

First, the analysis of the narratives found in the literature reveals the presence of tension as normal. Organizations are rife with tension and paradox (Schad et al. 2016) as is the interplay between work and family. The relationship may change but the tension is inescapable, defining the organization of work and family as processes imbued with a paradoxical ethos. Our analysis shows that the process changes without becoming non-paradoxical. For MOS this means that, instead of approaching workers from the angle of methodological individualism and the two realms of work and family as independent, it may be better to treat them as paradoxically interdependent or dialectically becoming a new synthesis. Instead of considering that the family is out of organizational bounds, family organization should be incorporated and treated as an inescapable stakeholder.

Second, the analysis indicates a progressive integration of work and non-work. The logic of separation is giving place to one of approximation, initially accelerated by dual career couples. If plausibility attached to the separation thesis, dual career couples made it less adequate. The COVID-19 pandemic collapsed work and family spaces and promoted hybrid workplaces, in which occupational responsibilities are partly assumed from home. Managing hybridity, for organizations that employ family members, will require more attention being given to the family organization of work. The integration of family organizations as stakeholders is particularly necessary. Our discussion thus invites MOS scholars to conceptualize family as a core stakeholder, a group that is affected by the organization’s actions but that, at present, lacks any forms of adequate representation. Family organization was obviously and indirectly (proposition 1) or directly (proposition 2) affected by employing organizations while the COVID-19 crisis, in the case of “non-essential workers”, literally integrated family and work organization. In this sense, family relations and their organization entered employing organizations virtually, through presence in Zoom meetings (proposition 3).

A key question for future research will be whether family organization is intruding on workplace organization or workplace organization is intruding on family organization? Our analysis, in summary, suggests that the dynamics of approximation point in the direction of hybrid models of work, indicating that the relationship between the organization of employment and family needs to be conceptualized as integral, rather than peripheral, to MOS.

### Implications for Theory

For theory we advance several possibilities that deserve further consideration. The conflict thesis, prevalent in meta-analytic work (e.g., Allen et al. 2012, 2015), captures the relationship between work and family as separate domains “mutually incompatible” (Greenhaus and Beutell 1985, p. 77). The pandemic crisis has blurred the boundaries between the two realms and challenged established theories. Regarding one of our research questions (*what does COVID-19 reveal about family-work tensions?*) we found that the emergence of the home office is creating liminal spaces (Söderlund and Borg 2018) with its own challenges, resulting from the dissolution of physical and psychological boundaries.

This change is expressed in the advent of “hybrid workers” and “hybrid work” arrangements that raise new challenges, as the traditional roles and spaces are being replaced with new notions of the meaning of the “office” (Davenport and Pearlson 1998). In other words, the unfolding changes, precipitated by the pandemic, constitute an invitation for revising the meaning of both the workplace and the homeplace and even creating a synthesis in the form of the home office.

The home office opens novel research avenues and stimulates new research streams in terms of law (e.g., can an employee’s home be legally framed as an extension of a distributed office?), control (e.g., how legitimate is the control of people in their private/family space?), and leadership (e.g., when do leaders lead when flex time becomes a norm?). These and other interrogations will be relevant to redraw the contours of work-family research and to re-conceptualize the topic as it is redefined by new technological possibilities.

### Implications for Practice

If organizations accept that the family is a legitimate stakeholder, then they need to consider the motives not only of their *employees as members* of family organizations but also the motives of the family organization and the ways its multiple members are relating to multiple organizations of work and education from home. Such an orientation will help organizations contribute to the common good (Schlag and Melé 2020). The structure and dynamics of families have implications for career progression, especially for working mothers (Benavides and Montes 2020), so addressing the family as an organization is an important endeavour, especially as it relates to other organizations, such as childcare provision, costs and regulation. Employing organizations may have to formulate policies to help their members to effectively manage work and family organization (Stein et al. 2021; Vaziri et al. 2020), a challenge already embraced by multinational companies regarding the expatriates’ family (e.g., “family is treated as a unit and included in the selection process” of expatriates; Anderson 2005, p. 567). Policies that can make role transitions less difficult will need to be implemented either by state, market or firm hierarchy.

While the mere recognition of family organization as a stakeholder will not change anything per se, if decisions take into consideration not the case of individual employees but also their family organization, more flexible solutions may emerge from consideration of the family (by soliciting, for instance, family perceptions of organizational cultures and practices, as is proposed by Li et al. 2021). The job of Human Resources departments will certainly become more complicated. Organizations may segment work to respond to different employee needs. Flexible policies may support such endeavour. Employing organizations may realize that neo-liberal instincts and a functioning care system that enables effective organization are inimical. They may even have to start lobbying for increased taxes if the state is to bear the burden of making family organization work for them.

More ethical decisions may also become normal; for example, if one family member is jobless, is it morally acceptable that the organization involves another family member in a downsizing (Leite 2012)? Or should it avoid causing extra-suffering and health issues through financial hardship (Pfeffer 2018)? These questions gain a new light if family organizations are taken as stakeholders. The question of representation arises: how do the demands and requirements of a multiplicity of family organizational forms become represented, ranked, and implemented? Questions for hiring are also raised. There are, again, good arguments both for and against recruiting family members to the same organization (Pfeffer and Salancik 2003; Southwest Airlines 1999). From the perspective of family organization, hiring a couple is not the same as hiring any two employees.

Given the demographic challenges confronting many societies, protecting families is critical to reverse trends and to prepare the future. It is thus possible to affirm that the implications of our discussion extend beyond the domain of MOS and will need to be considered by national states. The demographic crisis (Morgan 2003) constitutes a challenge for the sustainability of the welfare state in many parts of the world and is indicative of the need to think about the family as an organizational unit of analysis. Doing this may be indicative of the need to embrace flexible policies to allow employees to address parental duties or accommodation of aging parents. Articulating work and family may thus gain from the flexible solutions untapped by the pandemic. Organizations may learn from this experience to retain the elements that can have a positive impact on future working experiences.

### Limitations

Our discussion is bound by several limitations: the analysis combines past findings and emerging trends. The circumstances may shift and neutralize some of our conceptual propositions. We wrote this piece during the pandemic. Future developments will also be conditioned by variables pertaining to national culture, technology adoption and economic development. For example, it is hard to tell if diffuse cultures (Hampden-Turner and Trompenaars 2008), that more naturally allow work and private spheres to intersect, will be more accepting of this new hybrid reality or instead resist it because people miss their “family” at work. On the other hand, we are curious to observe whether specific cultures (Hampden-Turner and Trompenaars 2008), which tend to separate work and private life, will become more diffuse themselves, now that people were able to see that their fellow workers also have a family and share similar challenges at home. It is also possible that in cultures where family is regarded with higher importance (Jin et al. 2013; Zhao et al. 2019), organizations that treat family as a stakeholder get a higher level of social legitimacy.

The new-New Normal (Ahlstrom et al. 2020) that will emerge after this period may gain new shapes that might neutralize the trends proposed here. It is possible, in other words, that we have captured a moving target that will evanesce. It is admissible that some of the experiments will be suspended in the post-pandemic period. It is true that flexible designs propelled by new technologies were already under development (Hanelt et al. 2021), but the depth of their adoption in a post-pandemic world is unpredictable.

## Conclusion

The family is not often regarded as an organizational stakeholder, in both senses of the word “organizational”. Families have been conceptualized as external to organizations or as partly overlapping via their individual employees. From the stakeholder perspective (which considers an organization as set of relationships among groups that have a stake in the activities that make up the organization; Parmar et al. 2010), the absence of the family from the list of relevant stakeholders is surprising. From a stakeholder perspective and that of developing more humanistic management theories, the family dimension cannot be ignored. The COVID-19 pandemic catalysed change and created a superposition between family and work. This created an opportunity and the necessity to theorize the family as stakeholder. Anticipating that some forms of work adopted during the pandemic will persist, and that more people will work from home, there is space to accept family organization as an integral and legitimate organizational stakeholder.

## Appendix

Table 2

**Table 2:** Meta-analytical sources on work and family, organized chronologically

Sources	Number or articles included	Study variables	Main findings/conclusions
Kossek and Ozeki (1998)	27 published articles	Job satisfaction; Life satisfaction; WFC; FWC; Bidirectional work-family conflict; Men; Women; Married respondents; Dual-career couples.	Regardless of the type of measure used (bidirectional work-family conflict, work to family conflict, and family to wok conflict), a consistent negative relationship exists among all forms of work-family conflict and job-life satisfaction. This relationship is slightly less strong for family to work conflict.
Byron (2005)	60 articles	WIF; FIW; Work variables (job involvement, hours spent at work, work support, schedule flexibility, and job stress); Nonwork variables (family/nonwork involvement, hours of nonwork, family support, family stress, family conflict, number of children -living at home-, age of youngest child, marital status, spousal employment); Demographic variables (sex, income, coping style and skills); Moderators (percent female and percent parents in sample, and coding of antecedents).	There exists a differentiation between WIF and FIW. Employees seem to differentiate between the source, or direction, of interference, and the two types of interference appear to have different antecedents. However, some work and family factors can have simultaneously disruptive effects on employees’ work and family lives.
Mesmer-Magnus and Viswesvaran (2005)	20 studies	WFC; FWC; Job stressors; Non-work stressors; Supportive work environment; Organizational attachment; Organizational withdrawal behaviours; Job satisfaction; Life satisfaction; Health.	It is generally assumed that WFC and FWC are distinct forms of work/family conflict, as they originate from arguably separate life domains, and these articles report that the conclusion that these two types of conflict possess discriminant validity appears to be credible
Mesmer-Magnus and Viswesvaran (2006)	38 studies	Global work/family conflict; WFC; FWC; Family friendly work environments (FFWEs): (1) Work/family programs, policies, or benefits (flexibility and dependent care) and (2) Family-friendly culture (work/family culture, supervisor support, and co-worker support).	FFWEs play a relatively small role in worker reports of work/family conflict, hence, provide less assistance to workers in managing WFC than one may hope, as none explained more than 7 % of the variance in WFC. A family-friendly work culture seems most influential in reducing WFC. Spousal support and FFWEs explain different portions of variance in WFC, suggesting that FFWEs are uniquely valuable to workers in achieving work/ family balance.
Ford et al. (2007)	178 (published and unpublished) studies	WIF; FIW; Family satisfaction; Job satisfaction; Job involvement; Job stress; Work support; Work hours; Family hours; Family stress; Family support; Family conflict.	Stressors and sources of support that are specific to the work and the family domain are related to satisfaction outside of those domains. Overall, 7% of the variance in family satisfaction and 37% of WIF variance is related to variables within the work domain, whereas 7% of the variance in job satisfaction and 21% of FIW variance is explained by variables in the family domain. Stress from the work domain has the strongest relation of all of the variables examined with WIF and family satisfaction. Family stress and conflict are the strongest family domain correlates of job satisfaction, although these relations are not as strong as those between job stress and family satisfaction.
Michel and Hargis (2008)	167 studies	Work social support; Work involvement; Work role conflict; Work time demands; Work role ambiguity; WFC; Family satisfaction; Job satisfaction; Family social support; Family involvement; Family role conflict; Family time demands; Family role ambiguity; FWC.	Indirect effect work–family conflict models explain 2.20% and 6.20% of the variance in job and family satisfaction outcomes, whereas direct effect segmentation models explain 54.10% and 48.50% of the variance in job and family satisfaction outcomes
Michel et al. (2009)	211 studies	WIF; FIW; Work social support; Work involvement; Work role conflict; Work time demands; Work role conflicts; Work time demands; Work role ambiguity; Family social support; Family involvement; Family role conflict; Family time demands; Family role ambiguity; Job satisfaction; Family satisfaction; Life satisfaction.	Among the multiple full-range work-family conflict models and model linkages, direct effects drive work-family conflict models while indirect effects provide little incremental explanation in regard to satisfaction outcomes.
Hoobler et al. (2010)	90 studies	WFC; FWC; Work performance; Salary; Career satisfaction; Hierarchical level attained; Control variable: age.	Both WFC and FWC negatively impact self-rated as well as manager-rated work performance. WFC and FWC are negatively related to career satisfaction and hierarchical level attained. WFC is negatively related to salary, while FWC is positively related to salary.
McNall et al. (2010)	28 (published and unpublished) studies	WFE; FEW; Job satisfaction; Affective commitment; Turnover intentions; Family satisfaction; Life satisfaction; Physical/mental health.	WFE and FWE are positively associated with work-related outcomes (job satisfaction and affective commitment). When employees perceive that their work and family roles are enriching, they may reciprocate toward the organization with desired attitudes but not necessarily intentions to remain in the organization. WFE and FWE are positively linked to physical and mental health. The role from which enrichment originated is more strongly related to various outcomes than the role from which the enrichment is received, which is contrary to results in the work–family conflict literature. Thus, WFE has a stronger effect on work-related outcomes (job satisfaction and affective commitment); whereas FWE has a stronger effect on family satisfaction.
Michel et al. (2010)	129 studies	Work involvement; Work role conflict; Work time demands; WFC; Work social support; Work role ambiguity; Family involvement; Family role conflict; Family time demands; FWC; Family social support; Family role ambiguity.	Controlling for role involvement, work and family social support have the greatest effect on same-domain role stressors, which then have an effect on the cross-domain work–family conflict constructs. Controlling for work and family involvement, work and family social support are most related to same domain role conflict and role ambiguity. Subsequently, work role conflict and time demands are most related to WFC, while family role conflict and role ambiguity are most related to FWC.
Amstad et al. (2011)	98 articles	WIF; FIW; Different outcome variables (work-related outcomes, family-related outcomes, and domain-unspecific outcomes)	Work–family conflict affects well-being and behaviour in general, but also to family and working life’s well-being. WIF is more strongly associated with work-related than with family-related outcomes, while FIW is more strongly associated with family-related outcomes.
Kossek et al. (2011)	85 published and unpublished studies	WFC; Perceived Organizational Support; Perceived Work-Family Organizational support; Supervisor support; Supervisor Work-Family Support.	Work–family-specific support is more strongly related to work to family conflict than general support. Positive perceptions of general and work–family-specific supervisor indirectly relate to work–family conflict via organizational work–family support, that is, work–family-specific support plays a central role in individuals’ work–family conflict experiences.
Michel et al. (2011)	142 studies	WFC; FWC; Job stressors (work role conflict, work role ambiguity, work role overload); Work role involvement (job involvement, work interest/centrality); Work social support (organizational support, supervisor support, co-worker support); Work characteristics (organizational tenure, job tenure, type of job, current salary, task variety, job autonomy, schedule flexibility, family friendly organization); Family stressors (family role conflict, family role ambiguity, family role overload); Family role involvement (family involvement, family interest/centrality); Family social support (family support, spousal support); Family characteristics (working spouse, family income, family climate); Personality (Internal locus of control, negative affect/neuroticism); Demographic variables (marital status, parental status, gender).	Work role stressors, work role involvement, work social support, some work characteristics (task variety, job autonomy, family friendly organization), and personality are antecedents of WFC; while family role stressors (family stressors, role conflict, role ambiguity, role overload, time demands, parental demands, number of children/dependents), family social support, family climate, and personality are antecedents of FWC. In addition, work role stressors (job stressors, role conflict, role ambiguity, role overload) and work social support are predictors of FWC; while family role stressors, family involvement, family social support, and family climate are predictors of WFC
Shockley and Singla (2011)	132 (published and unpublished) studies	WIF; FIW; WEF; FEW; Job satisfaction; Family satisfaction; Control variables (job satisfaction and family satisfaction).	WIF is more strongly related to job satisfaction than family satisfaction, and FIW is more strongly related to family satisfaction than job satisfaction. Affective reactions to WFE occur mostly in the originating, rather than receiving, domain. With the exception WEF and job satisfaction, gender moderates all relationships such that the relationships are stronger when more females are in the sample.
Allen et al. (2012)	68 articles	WFC; Dispositions; Demographic moderators (percentage of male, parents and married participants in each sample)	Dispositions are important predictors of work–family conflict. In general, negative trait-based variables (e.g., negative affect and neuroticism) appear to make individuals more vulnerable to work–family conflict, while positive trait-based variables (e.g., positive affect and self-efficacy) appear to protect individuals from work–family conflict.
Allen et al. (2013)	58 articles	WFC; Flexible work arrangements; Moderators (percentage of male, parents, and married participants in each sample; and work hours of participants in the sample)	The relationship between flexible work arrangements and work–family conflict may be smaller than assumed because the direction of work–family conflict (WIF vs. FIW) and the specific form of flexibility (flexitime vs. flexplace; use vs. availability) make a difference in the effects found, but, overall, the significant effects are small in magnitude.
Butts et al. (2013)	57 studies (41 published and 16 unpublished studies)	Policy availability; Policy use; Family-supportive organization perceptions (FSOP); WFC; Work attitudes (job satisfaction, affective commitment, and intentions to stay); Sample characteristics (gender, marital status, responsibility for dependents); Study-level variables (publication status, geographic location).	Availability and use of work–family support policies exhibit small but favourable relationships with work attitudes, these relationships being stronger for availability than for use. Greater availability of work–family support policies is associated with higher FSOP and, in turn, related to more positive attitudes. Policy use is partially related to work attitudes through reduced WFC.
Allen et al. (2015)	20 studies (published and unpublished research)	WFC; National context	There are no significant differences in mean in WFC, while FWC is higher in more collectivistic cultures, in countries with a higher economic gender gap, and in countries other than the U.S.
Nohe et al. (2015)	30 relevant papers (17 published journal articles, 11 unpublished papers, and 2 conference papers)	WIF; FIW; Strain type; Work-specific strain (burnout, cynicism, depersonalization, disengagement, emotional exhaustion, irritation, need for recovery, and personal accomplishment).	WIF and FIW predict strain and strain predicts WIF and FIW. That is, there are reciprocal effects between WIF/FIW and strain. WIF has a stronger effect on work-specific strain than does FIW, which supports the matching hypothesis rather than the cross-domain perspective.
Fellows et al. (2016)	33 articles	Work–family conflict; Couple relationship quality; Moderators (gender, dual-earner versus single-earner status, parental status, region, and scale standardization).	Work–family conflict is associated with lower couple relationship quality. This relationship is stronger for single-earner couples and for couples in North America (versus those in Asia or Europe)
Litano et al. (2016)	40 (published and unpublished) studies	WIF; FIW; WFE; FWE; Cultural orientation (individualistic, collectivistic, power distance, autonomy); Publication source (published, unpublished); LMX measure (LMX-7 and LMX-MDM)	Leader–member exchange (LMX) is negatively related to WIF, and FIW, and positively linked to work-family enrichment, and family-work enrichment. Both contextual and methodological factors moderate the relationship between LMX and WIF. This study calls for incorporating established leadership theory into work-family research to better understand how and why leaders assist their employees in effectively managing work and family.
Shockley et al. (2017)	582 published papers, dissertations, and conference papers	Gender; WFC (WIF versus FIW); Moderators (dual-earner couples, parental status, full-time workers, same job types, cultural gender egalitarianism, date of publication); Mediators (work and family hours, work and family salience, boundary strength around work and family, coder training and process)	There is little evidence for substantial gender differences in WFC. Although the association between gender and WIF and FIW is statistically significant in the direction of women experiencing more conflict overall, the correlations are very small in magnitude and may be considered negligible for practical purposes. Interestingly, results differ somewhat by type of conflict; for example, men actually reported more time-based WIF than women, though the effect is still small.
French et al. (2018)	177studies (135 published, 34 dissertations, 7 conference presentations, and 1 unpublished data set)	WIF; FIW; Combined work support (organizational support, supervisor support, co-worker support, mixed supervisor/co-worker support, instrumental support, emotional support, mixed instrumental/emotional support, support behaviours, support perceptions, mixed support behaviour/perception); Combined family support (general family support, spouse support, instrumental support, emotional support, mixed instrumental/emotional support, support behaviours, support perceptions, mixed support behaviour/perception)	More social support emanating from the work domain consistently relates to less WIF and to less FIW. The magnitude of relationships between social support and work-family conflict varies as a function of social support domain, form, source, type, and national context.
Lapierre et al. (2018)	171 (published and unpublished) studies	WFE; FEW; Family-friendly organizational policies; Social support at work; Work autonomy; Social support from family; Work engagement	Some contextual and personal characteristics (e.g., social support at work, social support from family) are significantly associated with enrichment: those associated with work tend to have stronger relationships with WFE and those associated with family tend to have stronger relationships with FEW. However, some antecedents have significant relationships with both WFE and FEW. Work engagement mediates between several contextual characteristics and enrichment.
Xu et al. (2018)	250 articles (214 published and 36 unpublished papers)	WFC; WFE; Job satisfaction; Family satisfaction; Life satisfaction; Mental health	Employees from more individualistic and more developed countries are more sensitive to how work interferes with family life, whereas employees in less individualistic and less developed countries are more sensitive to how work provides material, social, and cognitive resources that help in the fulfilment of family roles.
Zhang et al. (2018)	67 articles	WFE; FWE; Work domain variables (job satisfaction, organizational commitment, turnover intention); resource consequences (work engagement, burnout); Performance variables (in-role performance, organizational citizenship, behavior); General well-being (overall health, life satisfaction, stress) and family related variables (family satisfaction, family performance); Moderators (gender, marital status, age, number of children, national culture, construct label)	The WFE has benefits for both work and family life, but it has stronger effects on within-domain consequences than cross-domain consequences. The relationship between FWE and job satisfaction is stronger in Eastern countries than Western countries.
Liao et al. (2019)	228 research papers	Work-family conflict; Work demand; Work control; Work role overload; Work hours; Job autonomy; Flexibility at work; Family demand; Family control; Family role overload; Family hours; Commitment to work; Commitment to family; Work performance; Family performance; Career satisfaction; Career development consequences	Work and family demands are positively related to WFC, while when individuals perceive that they have control over their work or family obligations, they are in a better position to retain or protect their limited resources for their dual roles in the work and family domains, leading to the mitigation of WFC. A high level of autonomy at work is negatively related to WFC, and hours spend at work has a positive relation with WFC. Role overload at both work and family are associated with WFC, while having flexibility from work schedule is negatively related to WFC. In addition, WFC is negatively related to employee career development outcomes.
Wong et al. (2020)	58 papers published	Work–life balance arrangement (WLBA: family-friendly policies, flexible working hours, incentive program, workplace health program, work-life balance program); Organizational performance (CM: career motivation, employee attendance, employment recruitment, employee retention, organizational commitment, productivity); Moderating variables (gender, sector, employee hierarchy, publication year, age, country of origin).	There exists a positive relationship between the work–life balance arrangement and organizational performance. Career motivation, employee attendance, employee recruitment, and employee retention are significantly associated with the work–life balance arrangement. The moderators affecting the relationship between the work–life balance arrangement and organizational performance were gender, sector, and employee hierarchy.
Xin et al. (2020)	71 papers	WFC; WIF; FIW; Year of data collection; Control variables (gender ratio, publication class, and region)	Social changes played an important role in changes of WFC because the increase in WFC scores among Chinese employees is associated with scores of six social indicators that might cause stress in workplace (the number of employees and number of college graduates) and stress in family (divorce rate, residents’ consumption level, elderly dependency ratio, and family size) of 5 years before and the year of data collection.
Zhao, Ghiselli et al. (2020)	57 studies	WIF; FIW; Demographics variables (age, education, gender, marital status, organizational tenure, and number of children); Work variables (work overload, work stress, job satisfaction, career satisfaction, turnover intention, intrinsic motivation, work performance, supervisor support, organizational support, affective commitment); non-work variables (positive affectivity, negative affectivity, emotional exhaustion, life satisfaction).	Work-family conflict significantly relates to employees’ work, family and life attitudes. However, there is no evidence indicating that work-family conflicts vary across demographic groups of employees. WIF and FIW are highly connected and influence each other.
Zhao, Wang et al. (2020)	42 articles	WIF; FIW; Job satisfaction; Life satisfaction; Gender; Organizational support; Number of children	Organizational support plays a critical role in helping employees release WFC and improve life satisfaction but not job satisfaction. The number of children is a key factor at the individual level on predicting WFC, whereas gender relates only to life satisfaction. There are asymmetric permeable roles of WFC dimensions (i.e., WIF and FIW) among work, family and life domains.
Hoobler et al. (2021)	55 articles	WF spillover; FW spillover; Mental health and well-being; Work strain; Home/family strain; Support from work; Job and career satisfaction; Work engagement; Life satisfaction; Home support; Autonomy; Support from home	When the magnitude of the relations between WFC and WFE and their common correlates (e.g., strain, support, and attitudes) in Africa with the West are compared, some differences in effect sizes are found. These differences could be due to African contexts, specifically the influence of the family system, economic insecurity, and blurring of roles.
Li et al. (2021)	98 studies (81 articles, 8 conference papers, 8 dissertations, and 1 book chapter)	Role senders’ work stressors; Role sender work attitudes; Role sender WFC; Role receiver psychological distress; Role receiver work attitudes; Role receiver family satisfaction.	There exists crossover of the role sender’s work stressors, work attitudes, and WFC to the role receiver’s psychological distress, family satisfaction, and work attitudes. These effects are mediated by the role sender’s positive social behaviour.
Matei et al. (2021)	36 articles	Own job/family demands; Own job/family resources; Own personal resources; Own WIF; Own well-being; Partner’s job/family demands; Partner’s job/family resources; Partner’s personal resources; Partner’s WIF; Partner’s well-being.	Both partners’ well-being measures have small proportions of shared variance. Little evidence of a crossover effect from one’s work-related variables toward the partners’ family-related well-being. Analyses do not support a crossover effect from one’s work–family interaction toward their partner’s well-being. New studies about how family-related resources and demands are related to wellbeing and personal resources in the crossover processes are necessary

FEW: Family enrichment of work; FIW: Family interference with work; FWC: Family-to-work conflict; WIF: Work interference with family; WEF: Work enrichment of family; WFC: Work-to-family conflict; WFE: Work-family enrichment.

Table 3

**Table 3:** Journals where meta-analytical sources on work and family were published

Journal title	Number of meta-analytical sources
Journal of Vocational Behavior	9
Journal of Applied Psychology	5
Journal of Organizational Behavior	2
Personnel Psychology	2
Africa Journal of Management	1
Applied Psychology: Health and Well-being	1
Career Development International	1
Frontiers in Psychology	1
International Journal of Contemporary Hospitality Management	1
International Journal of Environmental Research and Public Health	1
International Journal of Stress Management	1
Journal of Business and Psychology	1
Journal of Family and Economic Issues	1
Journal of Hospitality and Tourism Management	1
Journal of Labor Research	1
Journal of Management	1
Journal of Occupational Health Psychology	1
Leadership Quarterly	1
PsyCh Journal	1
Psychological Bulletin	1
TOTAL	34
